# Differential Diagnosis Tool for Parkinsonian Syndrome Using Multiple Structural Brain Measures

**DOI:** 10.1155/2013/571289

**Published:** 2013-03-20

**Authors:** Miho Ota, Yasuhiro Nakata, Kimiteru Ito, Kouhei Kamiya, Masafumi Ogawa, Miho Murata, Satoko Obu, Hiroshi Kunugi, Noriko Sato

**Affiliations:** ^1^Department of Mental Disorder Research, National Institute of Neuroscience, National Center of Neurology and Psychiatry, 4-1-1 Ogawa-Higashi, Kodaira, Tokyo 187-8502, Japan; ^2^Department of Radiology, National Center of Neurology and Psychiatry Hospital, 4-1-1, Ogawa-Higashi, Kodaira, Tokyo 187-8551, Japan; ^3^Department of Neurology, National Center of Neurology and Psychiatry Hospital, 4-1-1 Ogawa-Higashi, Kodaira, Tokyo 187-8551, Japan

## Abstract

Clinical differentiation of parkinsonian syndromes such as the Parkinson variant of multiple system atrophy (MSA-P) and cerebellar subtype (MSA-C) from Parkinson's disease is difficult in the early stage of the disease. To identify the correlative pattern of brain changes for differentiating parkinsonian syndromes, we applied discriminant analysis techniques by magnetic resonance imaging (MRI). T1-weighted volume data and diffusion tensor images were obtained by MRI in eighteen patients with MSA-C, 12 patients with MSA-P, 21 patients with Parkinson's disease, and 21 healthy controls. They were evaluated using voxel-based morphometry and tract-based spatial statistics, respectively. Discriminant functions derived by step wise methods resulted in correct classification rates of 0.89. When differentiating these diseases with the use of three independent variables together, the correct classification rate was the same as that obtained with step wise methods. These findings support the view that each parkinsonian syndrome has structural deviations in multiple brain areas and that a combination of structural brain measures can help to distinguish parkinsonian syndromes.

## 1. Introduction

Multiple system atrophy (MSA) is an adult-onset, sporadic, progressive neurodegenerative disease characterized by varying severity of parkinsonian features, and cerebellar ataxia, autonomic failure, and corticospinal disorders [1–4]. According to the clinical presentation, a parkinsonian type (MSA-P) and a cerebellar type of MSA (MSA-C) are distinguished [2]. Parkinson's disease (PD) is a progressive neurodegenerative movement disorder characterized by rigidity, tremor, and bradykinesia. Its prevalence increases with age, and it affects 1% of the population over age 65 [5].

PD and MSA are both alpha-synucleinopathies [6, 7]. Pathologically, in Parkinson's disease a massive loss of dopaminergic neurons in pars compacta of substantia nigra and intraneuronal Lewy bodies are present [8]. In MSA, neuronal loss and gliosis occur in the inferior olives, pons, transverse pontocerebellar fibers, cerebellum, substantia nigra, locus caeruleus, striatum, and the intermediolateral column of the spinal cord [9]. In MSA-P, the nigrostriatal system is the main site of pathology, but less severe degeneration can be widespread and usually includes the olivopontocerebellar system [9, 10]. In MSA-C, the olivopontocerebellar system is mainly affected along with loss of pontine neurons and transverse pontocerebellar fibres and atrophy of the middle cerebellar peduncles (MCPs) [9, 10]. Conventional magnetic resonance imaging (MRI) may also help distinguish the two forms of MSA. MSA-P shows “slit-like” marginal hyperintensity of the putamen [11]. Additionally, the “hot-cross bun” sign on T2-weighted and proton density images in the ventral pons has been reported to be related to MSA-C [12]. However, these MRI changes do not always occur [3]. Clinical differential diagnosis between PD and MSA is difficult in the early stage of the disease. Relevant works on the other tools like SPECT [13], transcranial brain sonography [14–16] and on optical coherence tomography [17] showed the effectiveness for differentiating PD from healthy volunteer. Additionally, combined use of 123I-(S)-2-hydroxy-3-iodo-6-methoxy-N-((1-ethyl-2-pyrrodinyl)-methyl) benzamide (IBZM), 123I-N-v-fluoropropyl-2b-carbomethoxy-3b-(4-iodophenyl)nortropan (FP-CIT), and meta-123I-iodobenzylguanidine (MIBG) distinguishes Parkinsons disease from atypical parkinsonian disorder, such as PSP and MSA with the accuracy of about 90% [18]. However, 3 SPECT/scintigraphy tests only for diagnosis are not practical.

Over the last few years, a number of MRI studies have focused on the identification of diagnostic markers helpful in the differential diagnosis of parkinsonian syndromes such as MSA, PD, and progressive supranuclear palsy (PSP) [19–22]. However, no studies have discriminated among PD, MSA-P, MSA-C, and healthy subjects simultaneously. In the present study, we hypothesized that we would be able to distinguish the PD and healthy subjects from the MSA subjects by using the infratentorial brain images and MSA-P and PD from the MSA-C and healthy subjects by using supratentorial images. The characteristic distribution of regional brain changes revealed by the gray matter volume data using the optimized, voxel-based morphometry (VBM) method and by the diffusion tensor imaging data using tract-based spatial statistics (TBSS) would have diagnostic values for discriminating such diseases.

## 2. Materials and Methods

### 2.1. Subjects

From November 2006 to November 2010, 200 consecutive patients whose chief complaints were parkinsonism underwent brain MRI at our institution. We excluded the patients with cerebrovascular diseases cortical infarctions, multiple lacunar lesions, leukoaraiosis, and other lesions above Fazekas's Grade 2 on T2-weighted images or fluid-attenuated inversion recovery (FLAIR) MRI [23], PSP, and corticobasal degeneration (CBD). Clinical diagnosis of PD and MSA was made according to the established consensus criteria [2, 24]. A probable clinical diagnosis was determined by two neurologist with more than 20 years of experience in the diagnosis of movement disorders (MO, MM). As a consequence, 18 consecutive patients with MSA-C, 12 patients with MSA-P, and 21 patients with PD were studied. Their characteristics are shown in Table 1. 24 out of 30 MSA patients were hospitalized for the detailed diagnosis, and the diagnosis of 30 MSA patients weas not changed during follow-up clinical assessments (mean period = 2.2 years). As for PD, the follow-up clinical assessments were conducted (mean period = 4.7 years) after the MRI imaging, and no additional pathology was detected. 21 age- and sex-matched healthy persons who demonstrated no current or past history of psychiatric illness or contact with psychiatric services were enrolled as controls. Participants were excluded if they had a prior medical history of central nervous system disease or severe head injury. The study protocol was approved by the ethics committee of the National Center of Neurology and Psychiatry, Japan. 

### 2.2. MRI Data Acquisition and Processing

MR studies were performed on a Magnetom Symphony 1.5 Tesla (Siemens, Erlangen, Germany). First, high-spatial-resolution, 3-dimensional (3D) T1-weighted images of the brain were obtained for morphometric study. The 3D T1-weighted images were scanned in the sagittal plane (TE/TR: 2.64/1580 ms; flip angle: 15°; effective slice thickness: 1.23 mm; slab thickness: 177 mm; matrix: 208 × 256; FOV: 256 × 315 mm^2^; acquisitions: 1) yielding 144 contiguous slices through the head. The raw 3D T1-weighted volume data were transferred to a workstation, and structural images were analyzed using an optimized VBM technique. Data were analyzed using Statistical Parametric Mapping 5 (SPM5) software (Welcome Department of Imaging Neuroscience, London, UK) running on MATLAB 7.0 (Math Works, Natick, MA). Images were processed using an optimized VBM script. The details of this process are described elsewhere [25]. First, each individual 3D-T1 image was normalized with the optimized VBM method. Normalized segmented images were modulated by multiplication with Jacobian determinants of the spatial normalization function to encode the deformation field for each subject, as tissue density changes in normal space. Gray matter volume and cerebrospinal fluid (CSF) volume images were smoothed using a 12 mm full width at half maximum of an isotropic Gaussian kernel. Diffusion tensor imaging (DTI) was then performed in the axial plane (TE/TR: 106/11,200 ms; FOV: 240 × 240 mm^2^; matrix: 96 × 96; 75 continuous transverse slices; slice thickness 2.5 mm with no interslice gap). Diffusion was measured along 64 noncollinear directions with the use of a diffusion-weighted factor *b* in each direction for 1000 s/mm^2^, and one image was acquired without use of a diffusion gradient. Recently, a novel processing technique has been published. In this technique, instead of trying to match each and every voxel in different subjects, DTI data is projected on a common pseudoanatomical skeleton and therefore does not need smoothing [26]. TBSS is available as part of the FSL 4.1 software package [27]. The TBSS script runs the nonlinear registration, aligning all fractional anisotropy (FA) images to the FMRIB58_FA template, which is supplied with FSL. The script then takes the target and affine-aligns it into a 1 × 1 × 1 mm MNI152 space. Once this is done, each subject's FA image has the nonlinear transform to the target and then the affine transform to the MNI152 space applied, resulting in a transformation of the original FA image into the MNI152 space. Next, TBSS creates the mean of all aligned FA images and applies thinning of the local tract structure to create a skeletonized mean FA image. In order to exclude areas of low FA and/or high intersubject variability from the statistical analysis, TBSS thresholds a mean FA skeleton with a certain FA value, typically 0.2. The resulting binary skeleton mask is a pseudoanatomical representation of the main fiber tracks and defines the set of voxels used in all subsequent processing. Finally, TBSS projects each subject's aligned FA image onto the skeleton. This results in skeletonized FA data. It is this file that feeds into the voxelwise statistics. In addition to DTI and 3D T1-weighted images, conventional axial T2-weighted images (TE/TR: 95/3500 ms; flip angle: 150°; slice thickness: 5 mm; intersection gap: 1.75 mm; matrix: 448 × 512; field of view (FOV): 210 × 240 mm^2^; acquisitions: 1) and fluid attenuation inversion recovery images in the axial plane (TE/TR: 101/8800 ms; flip angle: 150°; slice thickness: 3 mm; intersection gap: 1.75 mm; matrix: 448 × 512; FOV: 210 × 240 mm^2^; acquisition: 1) were acquired to exclude cerebrovascular disease or other diseases such as tumors, and hydrocephalus. On conventional MRI, no abnormal findings were detected in the brain of any subject. 

### 2.3. Statistical Analysis

We first evaluated the differences between the patients and healthy subjects using analysis of variance (ANOVA). These tests were performed with the SPSS software ver. 11 (SPSS Japan, Tokyo, Japan). There were no significant differences in age among patients and controls, but there were statistically significant differences in duration of illness between the patients with MSA-P and with PD (*P* = 0.012) and with MSA-C and with PD (*P* = 0.005).

**Figure 1:**
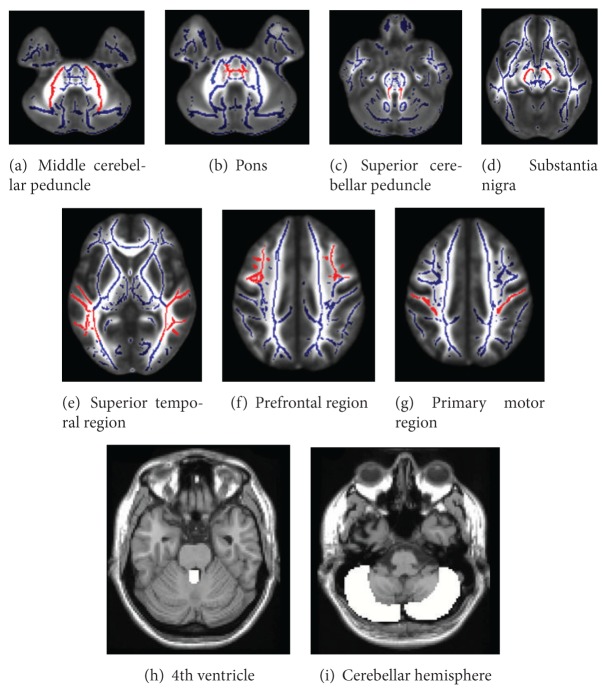
Locations of regions of interest. From (a) to (g) were put on the “FMRIB58_FA-skeleton_1 mm.nii” image, the anatomically standard image in FSL. Background fractional anisotropy image was the “MNI152_T1_1 mm.nii,” which was also the standard image in FSL. (h) and (i) were put on a “single_subj_T1.nii” image regarded as the anatomically standard image in SPM5.

The discriminant function analyses were then conducted to assess the ability of a combination of brain anatomical variables to distinguish between patients with MSA-C, MSA-P, Parkinson's disease, and controls. The independent variables were the volume data and fractional anisotropy value derived from the normalized individual image using the region of interests (ROI) method. ROIs were put on the “single_subj_T1.nii” image regarded as the anatomically standard image in SPM5, in the fourth ventricle, cerebellum hemisphere; these were derived from the WFU_PickAtlas, extension program of SPM5 [28, 29]. We also put ROIs on the “FMRIB58_FA-skeleton_1 mm.nii” image, which is the anatomically standard image in FSL, in the MCP, superior cerebellar peduncle (SCP), pons, substantia nigra, superior temporal white matter region, prefrontal white matter regions, and primary motor region where previous studies showed differences among the patients with MSA-C, MSA-P, PD, and controls (Figure 1) [20, 22, 30–37]. The value of a particular tissue was extracted using the software MarsBar [38], an extension program of SPM5.

The Box's *M* test confirmed the inequality of the group covariance matrices (Box-*M* = 76.63; *P* < 0.001). Discriminant functions were derived by step wise methods based on Mahalanobis' distance. The step wise selection criteria were decided by the overall multivariate *F* value of each variable to test differences between the patients and controls and to maximize the discriminant function between the groups. At the same time, we entered the two or three independent variables together and estimated the predictive power of the discriminant function.

## 3. Results

**Figure 2:**
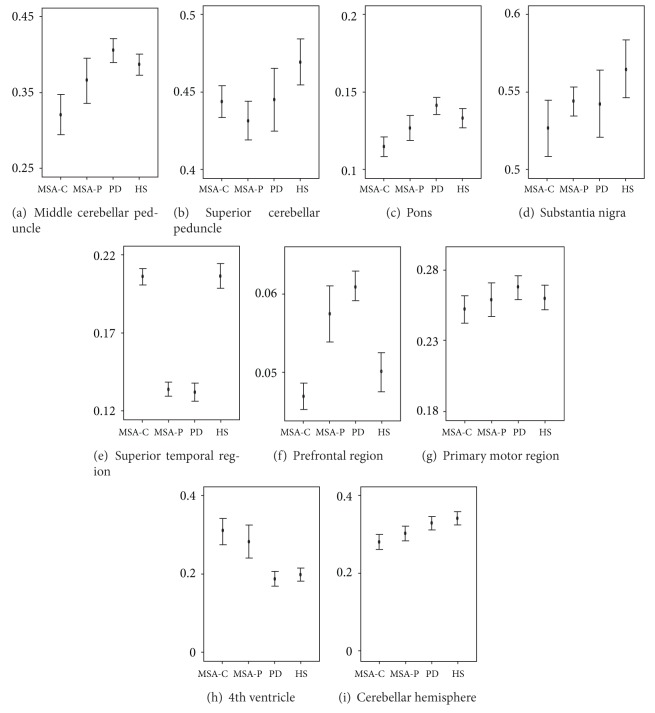
A graphic presentation of mean fractional anisotropy values in each region and mean volumes of 4th ventricular and cerebellum. MSA-C: multiple system atrophy with predominant cerebellar ataxia; MSA-P: multiple system atrophy with predominant parkinsonism; PD: Parkinson's disease; HS: healthy subjects.

We first calculated the volume and FA value from the spatially normalized images using ROIs. The mean values of these parameters are summarized in Table 2 and Figure 2. The mean FA value of the prefrontal region was too small to examine, so we did not evaluate the influence of the FA value in the prefrontal white matter region. We then conducted discriminant function analyses. The following five variables were entered in a step wise manner: fourth ventricle volume, substantia nigra, superior temporal, and prefrontal white matter region. The discriminant coefficients are shown in Table 3. The use of these variables resulted in correct classification rates of 0.89 (*χ* ^2^ = 294.66; df = 12; *P* < 0.001; Wilks' lambda = 0.012) (Table 4). 

**Figure 3:**
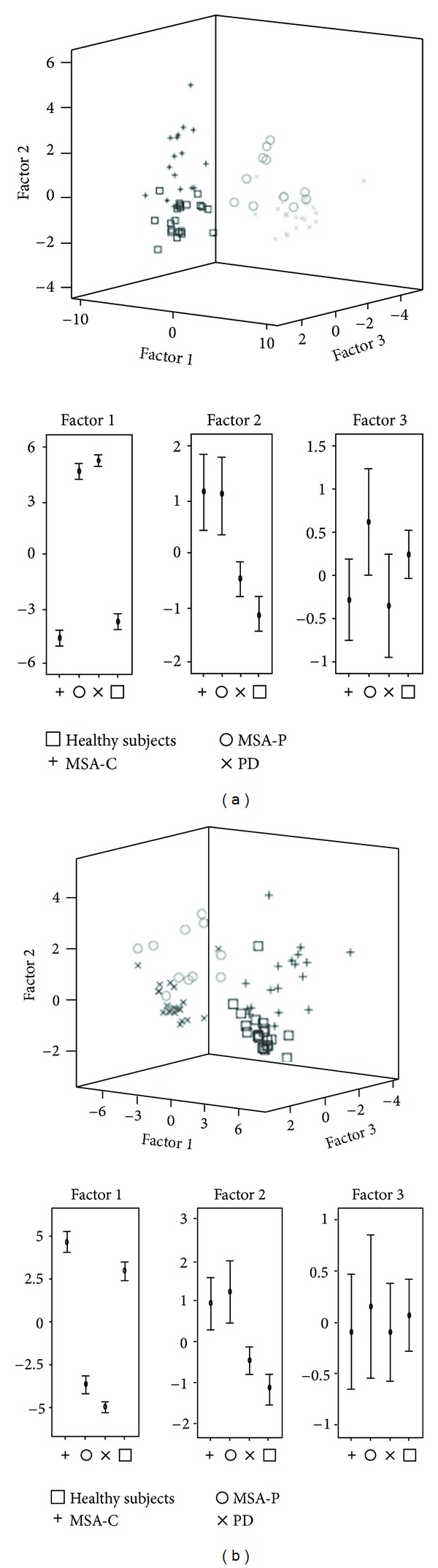
3-dimensional scattered plots showed the discriminant scores of each subject. These were calculated (a) by the analysis using step wise method and (b) using three independent variables together. Factors 1–3 were defined in Table 3.

The correct classification rates of each combination used to run the discriminant function analyses using two or three independent variables together are listed in Table 5. The highest correct classification rates were measured when we estimated the FA value of the “pons and superior temporal region” and “superior temporal region, MCP and fourth ventricle volume”, respectively. Table 3 shows the discriminant coefficients, and Table 4 shows the correct classification rates derived from the analyses using two or three independent variables together, in the same way.  Figure 3 shows the discriminant scores of each subject, calculated by the analysis using three independent variables together.

## 4. Discussion

 We found that the step wise discriminant function analysis identified with fairly good accuracy the combinations of ROIs that characterized brain anatomical features distinguishing the patients with MSA-C, MSA-P, PD, and healthy subjects, and that when discriminate analysis was conducted using the fourth ventricle volume and the FA value of MCP and superior temporal region as independent variables together, the correct classification rate was the same as that of step wise discriminant function analysis. 

One study showed that patients with MSA-C and MSA-P share similar diffusion tensor imaging features in the infratentorial region [22]. Furthermore, the combination of DTI metrics can be used to distinguish between patients with MSA and with PD. However, they could not differentiate the patients with PD from healthy subjects. This may be because they were focused on the infratentorial FA value and did not investigate the focal lesions related to parkinsonism. In this study, we used the FA value of the superior temporal regions, known to be impaired in PD as an independent variable, so we could discriminate the patients with PD from healthy subjects and with MSA-C from those with MSA-P [30–32, 34, 35].

One study reported discriminating patients with MSA-P, PD, PSP, and healthy subjects [20]. They indicated that investigating the degeneration of the MCP is useful for the in vivo differential diagnosis of MSA-P and PD. These results are congruent with our study. Establishing a means of differentiation using MR imaging would have potential therapeutic implications. 

 In this study, the participants with PD had a statistically longer duration of illness than those with MSA. It is known that MRI studies with PD show slight or no gray matter atrophy in early- to moderate-stage patients, whereas later-stage patients exhibited marked cortical atrophy [31]. We used the FA values for the independent variables to differentiate the patients with PD from others. White matter which appears normal on conventional MRI can show FA abnormalities, possibly permitting an earlier identification of the disease process which involves white matter tracts of the brain [33]. In addition, we successfully differentiated the patients with MSA-P from those with MSA-C using the same parameter. There is significant value to distinguishing these diseases using DTI metrics. 

All voxel-based analysis methods are susceptible to the effects of the spatial normalization transformation that registers images of different individuals. Regions in which this spatial transformation has relatively lower accuracy will tend to display artificially higher variability, which will adversely affect statistical significance. To date, TBSS is considered more robust and better suited for whole brain DTI data analysis. However, there are some limitations with the TBSS analysis. First, “FMRIB58_FA-skeleton_1 mm.nii” did not cover the thalamus and striatum, both of which have significant relationships with parkinsonian features [39]. A previous study demonstrated signal changes of the MR image after levodopa administration in an anatomical cluster which included the substantia nigra, tegmental ventral area subthalamic nucleus bilaterally, the principal origin, and first relay nuclei of projections in brain dopaminergic systems [40]. Therefore, we do not recommend using dopamine-rich regions such as the thalamus and striatum in discriminant function analysis for parkinsonian syndrome. Second, the predominant motor feature can change with time. The designation of MSA-P and MSA-C refers to the predominant feature at the time the patient is evaluated, and the predominant feature can change with time [2]. All of our MSA-C samples did not change the diagnosis to the MSA-P during follow-up clinical assessments; however, the discriminant method in this study would be fitted for the initial diagnosis. Third, our study is a small cross-sectional study, and we did not validate this discrimination method using another independent sample. MSA-P and MSA-C without cerebrovascular findings were so scarce, and we did not gather sufficient sample size. Further work with a large sample is required for the development of better discriminant capability and, if feasible, with data on another parkinsonism, PSP, would bring further clinical advantage.

## 5. Conclusions

Discriminant functions derived by step wise methods resulted in correct classification rates of 0.89. The present methods for automated analysis of morphometric data largely support findings from earlier studies using expert-guided ROIs or automated procedures. These findings support the view that each parkinsonian syndrome has structural deviations in multiple brain areas, and discriminant function analysis in this paper may provide objective biological information adjunct to the clinical diagnosis of parkinsonian syndromes.

## Figures and Tables

**Table 1:** Characteristics of the participants.

	MSA-C	MSA-P	PD	Normal volunteers
Mean age (years)	63.6 ± 7.8	61.9 ± 7.7	62.2 ± 7.0	62.3 ± 5.6
Sex (male : female)	7 : 11	6 : 6	10 : 11	11 : 10
Duration of illness (year)	3.9 ± 2.5	3.3 ± 2.6	6.8 ± 4.1	

MSA-C: cerebellar form of multiple system atrophy; MSA-P: parkinsonism forms of multiple system atrophy; PD: Parkinson's disease.

**Table 2:** Mean fractional anisotropy value in each region of interests and 4th ventricular and cerebellar volumes in patients with MSA-C, MSA-P, PD, and controls.

	MSA-C	MSA-P	PD	Normal
4th Vent_vol	0.31 ± 0.07	0.28 ± 0.07	0.19 ± 0.04	0.20 ± 0.04
Cerebellum_vol	0.28 ± 0.04	0.30 ± 0.03	0.33 ± 0.04	0.34 ± 0.04
MCP	0.32 ± 0.05	0.37 ± 0.05	0.41 ± 0.03	0.39 ± 0.03
SCP	0.44 ± 0.02	0.43 ± 0.02	0.45 ± 0.04	0.47 ± 0.03
Pons	0.12 ± 0.01	0.13 ± 0.01	0.14 ± 0.01	0.13 ± 0.01
SN	0.53 ± 0.04	0.54 ± 0.01	0.54 ± 0.05	0.56 ± 0.04
ST	0.21 ± 0.01	0.13 ± 0.01	0.13 ± 0.01	0.21 ± 0.02
PF	0.05 ± 0.00	0.06 ± 0.01	0.06 ± 0.00	0.05 ± 0.01
PM	0.20 ± 0.02	0.21 ± 0.02	0.22 ± 0.02	0.21 ± 0.02

4th Vent_vol: fourth ventricle volume; cerebellum_vol: cerebellum volume; MSA-C: cerebellar form of multiple system atrophy; MSA-P: parkinsonism forms of multiple system atrophy; MCP: middle cerebellar peduncle; SCP: superior cerebellar peduncle; PD: Parkinson's disease; SN: substantia nigra; ST: superior temporal region; PF: prefrontal region; PM: primary motor region.

**Table 3:** The coefficients of discriminant analysis.

		Factor 1	Factor 2	Factor 3
Stepwise method	4th Vent_vol	1.74	19.47	3.33
	SN	19.16	−9.81	38.32
	ST	−127.87	3.14	−32.07
	PF	196.3	80.22	−188.97
	(Constant)	0.66	−4.13	−5.99

Two independent variables analysis	Pons	−76.33	*69.41 *	(—)
	ST	*97.92 *	13.01	(—)
	(Constant)	−6.98	11.27	(—)

Three independent variables analysis	4th Vent_vol	1.14	*15.82 *	12.23
	MCP	−24.59	−5.39	*23.04 *
	ST	*100.68 *	−13.21	6.19
	(Constant)	−4.01	−3.68	−13.58

4th Vent_vol: fourth ventricle volume; MCP: middle cerebellar peduncle; SN: substantia nigra; ST: superior temporal region; PM: primary motor region.

**Table 4:** Classification results.

			Predicted group membership				
			MSA-C	MSA-P	PD	Control	Total
Stepwise method (88.9% of original grouped cases correctly classified)							

Original data	Count	MSA-C	15	0	0	3	18
		MSA-P	0	9	3	0	12
		PD	0	2	19	0	21
		Control	0	0	0	21	21
	%	MSA-C	83.3	0	0	16.7	100
		MSA-P	0	75.0	25.0	0	100
		PD	0	9.5	90.5	0	100
		Control	0.0	0	0	100.0	100

Two independent variables (84.7% of original grouped cases correctly classified)							

Original data	Count	MSA-C	15	0	0	3	18
		MSA-P	0	10	2	0	12
		PD	0	4	17	0	21
		Control	1	1	0	19	21
	%	MSA-C	83.3	0.0	0	16.7	100
		MSA-P	0	83.3	16.7	0	100
		PD	0	19.0	81.0	0	100
		Control	4.8	4.8	0.0	90.5	100

Three independent variables (88.9% of original grouped cases correctly classified)							

Original data	Count	MSA-C	14	0	0	4	18
		MSA-P	0	10	2	0	12
		PD	0	1	20	0	21
		Control	0	1	0	20	21
	%	MSA-C	77.8	0.0	0	22.2	100
		MSA-P	0	83.3	16.7	0	100
		PD	0	4.8	95.2	0	100
		Control	0.0	4.8	0.0	95.2	100

MSA-C: cerebellar form of multiple system atrophy; MSA-P: parkinsonism forms of multiple system atrophy; PD: Parkinson's disease.

**Table:** (a) Two independent variables

	4th Vent_vol	Cerebellum_vol	MCP	SCP	Pons	SN	ST	PM
4th Vent_vol		0.514	0.583	0.611	0.583	0.556	0.819	0.431
Cerebellum_vol			0.528	0.556	0.514	0.458	0.722	0.458
MCP				0.708	0.472	0.667	0.833	0.528
SCP					0.681	0.528	0.736	0.542
Pons						0.667	0.847	0.514
SN							0.722	0.583
ST								0.653
PM								

4th Vent_vol: fourth ventricle volume; cerebellum_vol: cerebellum volume; MCP: middle cerebellar peduncle; SCP: superior cerebellar peduncle; SN: substantia nigra; ST: superior temporal region; PM: primary motor region.

**Table:** (b) Three independent variables

								Accuracy
4th Vent_vol	Cerebellum_vol	MCP						0.556
4th Vent_vol	Cerebellum_vol		SCP					0.681
4th Vent_vol	Cerebellum_vol			Pons				0.569
4th Vent_vol	Cerebellum_vol				SN			0.583
4th Vent_vol	Cerebellum_vol					ST		0.861
4th Vent_vol	Cerebellum_vol						PM	0.583
4th Vent_vol		MCP	SCP					0.708
4th Vent_vol		MCP		Pons				0.542
4th Vent_vol		MCP			SN			0.653
4th Vent_vol		MCP				ST		*0.889 *
4th Vent_vol		MCP					PM	0.569
4th Vent_vol			SCP	Pons				0.722
4th Vent_vol			SCP		SN			0.625
4th Vent_vol			SCP			ST		0.833
4th Vent_vol			SCP				PM	0.681
4th Vent_vol				Pons	SN			0.681
4th Vent_vol				Pons		ST		0.889
4th Vent_vol				Pons			PM	0.597
4th Vent_vol					SN	ST		0.875
4th Vent_vol					SN		PM	0.611
4th Vent_vol						ST	PM	0.861
	Cerebellum_vol	MCP	SCP					0.681
	Cerebellum_vol	MCP		Pons				0.486
	Cerebellum_vol	MCP			SN			0.639
	Cerebellum_vol	MCP				ST		0.833
	Cerebellum_vol	MCP					PM	0.528
	Cerebellum_vol		SCP	Pons				0.625
	Cerebellum_vol		SCP		SN			0.583
	Cerebellum_vol		SCP			ST		0.764
	Cerebellum_vol		SCP				PM	0.583
	Cerebellum_vol			Pons	SN			0.653
	Cerebellum_vol			Pons		ST		0.833
	Cerebellum_vol			Pons			PM	0.528
	Cerebellum_vol				SN	ST		0.792
	Cerebellum_vol				SN		PM	0.583
	Cerebellum_vol					ST	PM	0.792
		MCP	SCP	Pons				0.681
		MCP	SCP		SN			0.694
		MCP	SCP			ST		0.819
		MCP	SCP				PM	0.681
		MCP		Pons	SN			0.694
		MCP		Pons		ST		0.806
		MCP		Pons			PM	0.486
		MCP			SN	ST		0.833
		MCP			SN		PM	0.625
		MCP				ST	PM	0.819
			SCP	Pons	SN			0.667
			SCP	Pons		ST		0.819
			SCP	Pons			PM	0.681
			SCP		SN	ST		0.792
			SCP		SN		PM	0.611
			SCP			ST	PM	0.722
				Pons	SN	ST		0.819
				Pons	SN		PM	0.694
				Pons		ST	PM	0.819
					SN	ST	PM	0.764

## References

[1] Geser F, Wenning GK, Seppi K (2006). Progression of multiple system atrophy (MSA): a prospective natural history study by the European MSA Study Group (EMSA SG). *Movement Disorders*.

[2] Gilman S, Wenning GK, Low PA (2008). Second consensus statement on the diagnosis of multiple system atrophy. *Neurology*.

[3] Quinn N (1986). Multiple system atrophy—the nature of the beast. *Journal of Neurology, Neurosurgery & Psychiatry*.

[4] Wenning GK, Tison F, Shlomo YB, Daniel SE, Quinn NP (1997). Multiple system atrophy: a review of 203 pathologically proven cases. *Movement Disorders*.

[5] Aarsland D, Andersen K, Larsen JP, Lolk A, Nielsen H, Kragh-Sørensen P (2001). Risk of dementia in Parkinson’s disease: a community-based, prospective study. *Neurology*.

[6] Spillantini MG, Crowther RA, Jakes R, Cairns NJ, Lantos PL, Goedert M (1998). Filamentous *α*-synuclein inclusions link multiple system atrophy with Parkinson’s disease and dementia with Lewy bodies. *Neuroscience Letters*.

[7] Wakabayashi K, Yoshimoto M, Tsuji S, Takahashi H (1998). *α*-synuclein immunoreactivity in glial cytoplasmic inclusions in multiple system atrophy. *Neuroscience Letters*.

[8] Fearnley JM, Lees AJ (1991). Ageing and Parkinson’s disease: substantia nigra regional selectivity. *Brain*.

[9] Wenning GK, Tison F, Elliott L, Quinn NP, Daniel SE (1996). Olivopontocerebellar pathology in multiple system atrophy. *Movement Disorders*.

[10] Kume A, Takahashi A, Hashizume Y (1993). Neuronal cell loss of the striatonigral system in multiple system atrophy. *Journal of the Neurological Sciences*.

[11] Bhattacharya K, Saadia D, Eisenkraft B (2002). Brain magnetic resonance imaging in multiple-system atrophy and Parkinson disease: a diagnostic algorithm. *Archives of Neurology*.

[12] Savoiardo M, Strada L, Girotti F (1990). Olivopontocerebellar atrophy: MR diagnosis and relationship to multisystem atrophy. *Radiology*.

[13] Spiegel J, Möllers MO, Jost WH (2005). FP-CIT and MIBG scintigraphy in early Parkinson’s disease. *Movement Disorders*.

[14] Becker G, Seufert J, Bogdahn U, Reichmann H, Reiners K (1995). Degeneration of substantia nigra in chronic Parkinson’s disease visualized by transcranial color-coded real-time sonography. *Neurology*.

[15] Busse K, Heilmann R, Kleinschmidt S (2012). Value of combined midbrain sonography, olfactory and motor function assessment in the differential diagnosis of early Parkinson's disease. *Journal of Neurology, Neurosurgery & Psychiatry*.

[16] Izawa Okawa M, Miwa H (2012). Transcranial sonography findings in Parkinson's disease. *Brain Nerve*.

[17] Albrecht P, Müller AK, Südmeyer M (2012). Optical coherence tomography in parkinsonian syndromes. *PLoS ONE*.

[18] Südmeyer M, Antke C, Zizek T (2011). Diagnostic accuracy of combined FP-CIT, IBZM, and MIBG scintigraphy in the differential diagnosis of degenerative parkinsonism: a multidimensional statistical approach. *Journal of Nuclear Medicine*.

[19] Gama RL, Távora DFG, Bomfim RC, Silva CE, de Bruin VM, de Bruin PF (2010). Morphometry MRI in the differential diagnosis of parkinsonian syndromes. *Arquivos de Neuro-Psiquiatria*.

[20] Nicoletti G, Lodi R, Condino F (2006). Apparent diffusion coefficient measurements of the middle cerebellar peduncle differentiate the Parkinson variant of MSA from Parkinson’s disease and progressive supranuclear palsy. *Brain*.

[21] Quattrone A, Nicoletti G, Messina D (2008). MR imaging index for differentiation of progressive supranuclear palsy from Parkinson disease and the Parkinson variant of multiple system atrophy. *Radiology*.

[22] Schocke MFH, Seppi K, Esterhammer R (2002). Diffusion-weighted MRI differentiates the Parkinson variant of multiple system atrophy from PD. *Neurology*.

[23] Fazekas F, Chawluk JB, Alavi A (1987). MR signal abnormalities at 1.5 T in Alzheimer’s dementia and normal aging. *American Journal of Roentgenology*.

[24] Gibb WRG, Lees AJ (1998). The relevance of the Lewy body to the pathogenesis of idiopathic Parkinson’s disease. *Journal of Neurology, Neurosurgery & Psychiatry*.

[25] Good CD, Johnsrude I, Ashburner J, Henson RNA, Friston KJ, Frackowiak RSJ (2001). Cerebral asymmetry and the effects of sex and handedness on brain structure: a voxel-based morphometric analysis of 465 normal adult human brains. *NeuroImage*.

[26] Smith SM, Jenkinson M, Johansen-Berg H (2006). Tract-based spatial statistics: voxelwise analysis of multi-subject diffusion data. *NeuroImage*.

[27] Smith SM, Jenkinson M, Woolrich MW (2004). Advances in functional and structural MR image analysis and implementation as FSL. *NeuroImage*.

[28] Maldjian JA, Laurienti PJ, Kraft RA, Burdette JH (2003). An automated method for neuroanatomic and cytoarchitectonic atlas-based interrogation of fMRI data sets. *NeuroImage*.

[29] Maldjian JA, Laurienti PJ, Burdette JH (2004). Precentral gyrus discrepancy in electronic versions of the Talairach atlas. *NeuroImage*.

[30] Beyer MK, Janvin CC, Larsen JP, Aarsland D (2007). A magnetic resonance imaging study of patients with Parkinson’s disease with mild cognitive impairment and dementia using voxel-based morphometry. *Journal of Neurology, Neurosurgery & Psychiatry*.

[31] Borghammer P, Østergaard K, Cumming P (2010). A deformation-based morphometry study of patients with early-stage Parkinson’s disease. *European Journal of Neurology*.

[32] Gerhard A, Pavese N, Hotton G (2006). In vivo imaging of microglial activation with [11C](R)-PK11195 PET in idiopathic Parkinson’s disease. *Neurobiology of Disease*.

[33] Prakash N, Hageman N, Hua X, Toga AW, Perlman SL, Salamon N (2009). Patterns of fractional anisotropy changes in white matter of cerebellar peduncles distinguish spinocerebellar ataxia-1 from multiple system atrophy and other ataxia syndromes. *NeuroImage*.

[34] Ramírez-Ruiz B, Martí MJ, Tolosa E (2005). Longitudinal evaluation of cerebral morphological changes in Parkinson's disease with and without dementia. *Journal of Neurology*.

[35] Specht K, Minnerop M, Abele M, Reul J, Wüllner U, Klockgether T (2003). In vivo voxel-based morphometry in multiple system atrophy of the cerebellar type. *Archives of Neurology*.

[36] Specht K, Minnerop M, Müller-Hübenthal J, Klockgether T (2005). Voxel-based analysis of multiple-system atrophy of cerebellar type: complementary results by combining voxel-based morphometry and voxel-based relaxometry. *NeuroImage*.

[37] Tzarouchi LC, Astrakas LG, Konitsiotis S (2010). Voxel-based morphometry and voxel-based relaxometry in parkinsonian variant of multiple system atrophy. *Journal of Neuroimaging*.

[38] Brett M, Anton J-L, Valabregue R, Poline J-B Region of interest analysis using an SPM toolbox.

[39] Remy P, Doder M, Lees A, Turjanski N, Brooks D (2005). Depression in Parkinson’s disease: loss of dopamine and noradrenaline innervation in the limbic system. *Brain*.

[40] Salgado-Pineda P, Delaveau P, Falcon C, Blin O (2006). Brain T1 intensity changes after levodopa administration in healthy subjects: a voxel-based morphometry study. *British Journal of Clinical Pharmacology*.

